# DNA damage induced by cis- and carboplatin as indicator for in vitro sensitivity of ovarian carcinoma cells

**DOI:** 10.1186/1471-2407-9-359

**Published:** 2009-10-10

**Authors:** Florian T Unger, Hermann A Klasen, Garri Tchartchian, Rudy L de Wilde, Irene Witte

**Affiliations:** 1 Institute for Biology and Environmental Sciences, Faculty V, University of Oldenburg, Oldenburg, Germany; 2 Department of Oncology (HAK) and Gynaecology (HR, RdW), Pius-Hospital, Oldenburg, Germany; 3 Department of Gynaecology and Obstetrics Clinic, Pius Hospital, Oldenburg, Germany

## Abstract

**Background:**

The DNA damage by platinum cytostatics is thought to be the main cause of their cytotoxicity. Therefore the measurement of the DNA damage induced by cis- and carboplatin should reflect the sensitivity of cancer cells toward the platinum chemotherapeutics.

**Methods:**

DNA damage induced by cis- and carboplatin in primary cells of ovarian carcinomas was determined by the alkaline comet assay. In parallel, the reduction of cell viability was measured by the fluorescein diacetate (FDA) hydrolysis assay.

**Results:**

While in the comet assay the isolated cells showed a high degree of DNA damage after a 24 h treatment, cell viability revealed no cytotoxicity after that incubation time. The individual sensitivities to DNA damage of 12 tumour biopsies differed up to a factor of about 3. DNA damage after a one day treatment with cis- or carboplatin correlated well with the cytotoxic effects after a 7 day treatment (r = 0,942 for cisplatin r = 0.971 for carboplatin). In contrast to the platinum compounds the correlation of DNA damage and cytotoxicity induced by adriamycin was low (r = 0,692), or did not exist for gemcitabine.

**Conclusion:**

The measurement of DNA damage induced by cis- and carboplatin is an accurate method to determine the in vitro chemosensitivity of ovarian cancer cells towards these cytostatics, because of its quickness, sensitivity, and low cell number needed.

## Background

Cis- and carboplatin used in the standard chemotherapy of ovarian carcinomas possess DNA damaging properties. The DNA damage by the platinum compounds is thought to be the main cause of their cytotoxicity [1] whereby the DNA damages result in inhibition of growth and subsequently in apoptosis and necrosis [2]. It was shown that the platinum-DNA adducts directly correlate with the disease response [3]. Therefore, the measurement of the DNA damage induced by cis- and carboplatin in ovarian carcinoma cells should reflect the sensitivity of these cells toward the platinum chemotherapeutics.

DNA damage in single cells can be determined by several standard methods like measuring chromosome aberrations (CA), micronucleus test (MNT), or the comet assay. In contrast to the comet assay, the CA test and the MNT can only be used for dividing cells. Because of the low division rate of primary ovarian cancer cells in vitro the comet assay seems to be the method of choice as a rapid and highly sensitive test for the determination of DNA damages in cells of ovarian cancer biopsies. In the alkaline comet assay directly induced single strand breaks can be detected as well as alkaline labile sites and enzymatically induced strand breaks by DNA repair endonucleases [4,5]. DNA crosslinks of cis- and carboplatin can be indirectly determined by introducing single strand breaks in platinum treated DNA via x-rays or strand breaking chemicals like methyl methanesulfonate (MMS) [6]. The reduction of the single strand breaks provoked by e.g. MMS alone quantitatively reflects the crosslinks induced by the platinum compounds. Even though the portion of interstrand crosslinks is only 1-2% of all the DNA damage induced by cis- and carboplatin [1,7] the constant ratio of the adducts allows the representative determination of all DNA damages by measuring the interstrand crosslinks only.

If DNA damage induced by cis- and carboplatin is the main cause for cytotoxicity a strong correlation between both parameters is expected. Under these conditions, a correlation of DNA damage and clinical outcome is expected too because a correlation between cytotoxicity and clinical outcome was repeatedly shown [8-12]. Therefore, we determined DNA damage as well as cytotoxicity after treatment of the isolated cells from ovarian cancer biopsies with cis- and carboplatin. In addition to the platinum compounds, we also determined the DNA damage and cytotoxicity provoked by adriamycin and gemcitabine, two chemotherapeutics also used in the treatment of recurrent ovarian carcinomas. It is known that both compounds possess a broad spectrum of cell damaging properties besides the DNA damage [13,14]. Here, we expected a lower correlation between DNA damage and cell viability compared to the platinum compounds. Last but not least, we determined the variability in the sensitivity of 12 biopsies of ovarian carcinomas towards cis- and carboplatin. The problem of resistance of ovarian cancer cells especially after a first line chemotherapy has been described [8]. For the oncologist the knowledge of platinum sensitivity of the individual tumours would be important to take his choice of cytostatics for the second line therapy.

The aim of this study was to elucidate if the determination of platinum induced DNA damage in the comet assay, is an appropriate tool to rapidly recognize the individual platinum sensitivity of cells from ovarian cancer patients.

## Methods

### Chemicals

Gemcitabine (GEMZAR^®^, 200 mg) was obtained from Lilly, Gießen, F.R.G., cisplatin (PLATINEX^®^, 0.5 mg/ml) from Bristol-Myers Squibb, München, F.R.G, adriamycin (DOXORUBICIN^®^, 2 mg/ml) from Hexal, Holzkirchen, Germany and carboplatin (Carbomedac^®^, 10 mg/ml) from Medac, Wedel, Germany. Methyl methanesulfonate was purchased from Fluka, Buchs, Switzerland. Collagenase A, DNase and dispase II were provided by Roche, Mannheim, F.R.G. Cell culture medium (quantum 333) was purchased from PAA, Pasching, Austria.

### Isolation of cancer cells and cell culture

Cancer biopsies were obtained from patients with primary or recurrent epithelial ovarian cancer, identified by histological and cytological examination. Specimens were obtained according to protocols approved by the hospital institutional Review Board and the institutional ethical guidelines, after patient informed consent was received. For transport lasting 30-90 min, specimens were placed into sterile flasks containing medium enriched with 5% penicillin/streptomycin. Under sterile conditions biopsies were dispersed in 0.5 - 2.0 mm^3 ^fragments after excision of fat tissue. Fragments were then dissociated to a cell suspension of single cells by incubation in 5-10 ml enzymatic dissociation solution (collagenase/DNase) for 1-2 hours at 37°C on a shaker at 800 rpm. Small cell aggregates were excluded by filtration (0.2 μm pore size). Thereafter erythrocyte lysis reagent was repeatedly added to eliminate erythrocytes. Cells were washed twice with medium and seeded for the experiment. The viability of the isolated cells was 85-95% measured by trypane blue dye exclusion.

### Cytotoxicity measurement (FDA assay)

The FDA assay is based on measurement of fluorescence generated by enzymatic hydrolysis of fluorescein diacetate (FDA) to fluorescein which is retained within the living cells. The FDA assay was performed according to Rotman and Papermaster [15], modified by Larsson et al.[9]. Treatment with the chemotherapeutics dissolved in Quantum 333 occured 36 hours after seeding of 6000 cells/well of a 96 well plate at 37°C, 5% CO_2_/95% air with more than 95% humidity. Five concentrations of each drug with four replicates were tested. Peak serum concentrations were used (cisplatin: 100% peak serum concentration = 12,6 μM, carboplatin: 100% peak serum concentration = 42,5 μM, adriamycin: 100% peak serum concentration = 919,9 nM, gemcitabine: 100% peak serum concentration = 80,8 μM). After an exposure of 7 days the drug containing medium was removed and 200 μl/well FDA solution (1 μg/ml in phosphate buffered saline) were added for one hour and incubated at 37°C, 5% CO_2_/95% air with more than 95% humidity. Thereafter the fluorescence was measured with a microplate fluorescence reader (FLUOstar, Offenbach, F.R.G.) with an excitation of 485 nm and an emission of 538 nm. Fluorescence data were analyzed using SPSS 11.5.1.

### Genotoxicity measurement (Comet assay)

The comet assay was performed according to Tice et al. [16]. Briefly, 4.000-10.000 cells/well were seeded into 24-well plates. 36 hours after seeding, the cells were treated with the chemotherapeutic agents. Drug incubation was conducted for 24 hours. DNA damage induced by the crosslinkers cis- and carboplatin was indirectly measured in the comet assay. Therefore, it was detected by adding the DNA strand breaking agent methyl methanesulfonate (MMS, 0.9 mM) during the last hour of incubation. The reduction of DNA strand breaks induced by MMS quantitatively reflects the DNA crosslinks induced by cis- and carboplatin [6]. After chemical treatment, cells were washed twice, trypsinized and resuspended in 40 μl ice-cold PBS-buffer. A volume of 20 μl of the resuspended cells was mixed with 80 μl 0.5% low melting agarose at 37°C and applied to pretreated microscope slides.

Pretreatment of slides involved coating with 1.5% agarose, diluted in Ca^2+ ^and Mg^2+^- free PBS (phosphate buffered saline, pH 7,4). Each concentration was performed in duplicate. The slides mounted with cells were covered with coverslips and kept in the refrigerator for 3-5 min to solidify the low melting agarose. The following steps were performed under dim-light to prevent additional UV-induced DNA damage. After removing the coverslips, slides were immersed in 4°C cold lysing solution pH 10.0 (2,5 M NaCl, 100 mM EDTA, 10 mM Tris, 1% N-lauroyl sarcosine, 1% Triton X100, 10% DMSO; the last two compounds were added freshly). Slides were kept at 4°C for 1 h. After lysis, the slides were placed on a horizontal electrophoresis box. The unit was filled with freshly prepared alkaline buffer (300 mM NaOH, 1 mM EDTA, pH 13), until slides were completely covered with buffer. After an incubation for 40 min at 4°C in alkaline buffer, to allow DNA unwinding and DNA breakage at alkali labile sites, DNA electrophoresis was performed in an ice bath at 25 V and 300 mA for 20 min. After electrophoresis, the slides were covered with neutralization buffer (0.4 M Tris HCl, pH 7.5) for 5 min. This step was repeated twice. Thereafter, the slides were briefly dipped into water and dried by air overnight. Finally, 40 μl ethidium bromide (20 μg/ml) was added to each slide. Slides were covered with a coverslip and kept for 5 min in the dark for DNA staining. DNA migration was analyzed by fluorescence microscopy (Nikon, Eclipse E600W). The tail moment (tm) was determined using the software "Lucia comet assay single stain" (Nikon). The tm considers the length of the tail as well as the intensity of the fluorescence staining of the tail, compared to the staining of the comet core. From each concentration, 50 randomly selected cells (25 cells from each of two duplicate slides) were analyzed. For statistical analysis, Mann-Whitney-U-Test was performed using SPSS 11.5.1

## Results

Freshly isolated cells of biopsies from patients with ovarian carcinomas were examined in their sensitivities towards cis- and carboplatin. To optimize the experimental conditions time and concentration dependent cyto- and genotoxicity was measured.

**Figure 1:**
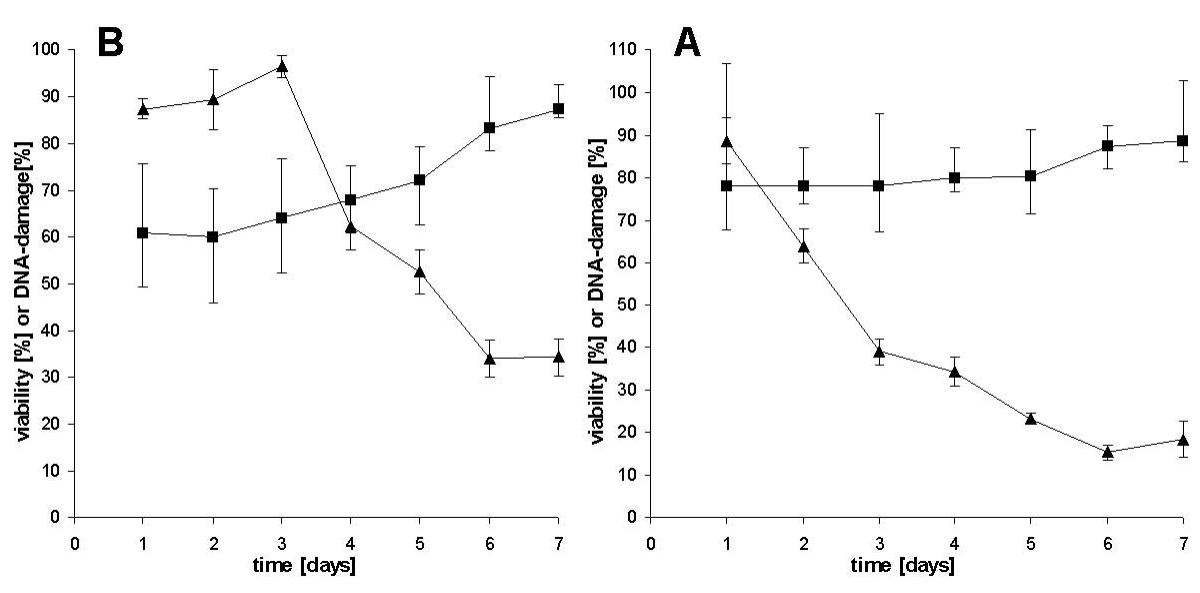
**Time dependent DNA damage (black square) presented as median with first and third quartile and cytotoxicity (black triangle) presented as mean with standard deviation induced by 100% SPK cisplatin (A), or 100% SPK carboplatin (B) in primary ovarian carcinoma cells**. DNA damage is shown as reduction of MMS induced tail moments (tm) by the platinum compound (50 comets/sample evaluated). MMS alone induced a tail moment ranging from 80-90. The platinum compounds alone induced no tail moments.

The time dependent geno- and cytotoxic effect of carbo- and cisplatin was determined up to 7 days. The results for cisplatin are shown in Fig. 1A, and for carboplatin in Fig. 1B. After a 24 h incubation with cisplatin nearly 80% of the measurable DNA damage had occurred. It increased to 87% at day 7. The DNA damage of 60% induced by carboplatin was somewhat lower than that of cisplatin after a one day incubation. After day 7 genotoxicity was similar for both compounds. The high degree of DNA damage after a 24 h incubation was nearly non-cytotoxic (Fig. 1), but the cisplatin cytotoxicity increased at day 2, and at day 6 a reduction of the cell viability of 85% was observed (Fig 1A). Carboplatin was less toxic than cisplatin. Cytotoxicity was observed at day 4 and reached a reduction of cell viability to 35% at day 6 (Fig. 1B).

**Figure 2:**
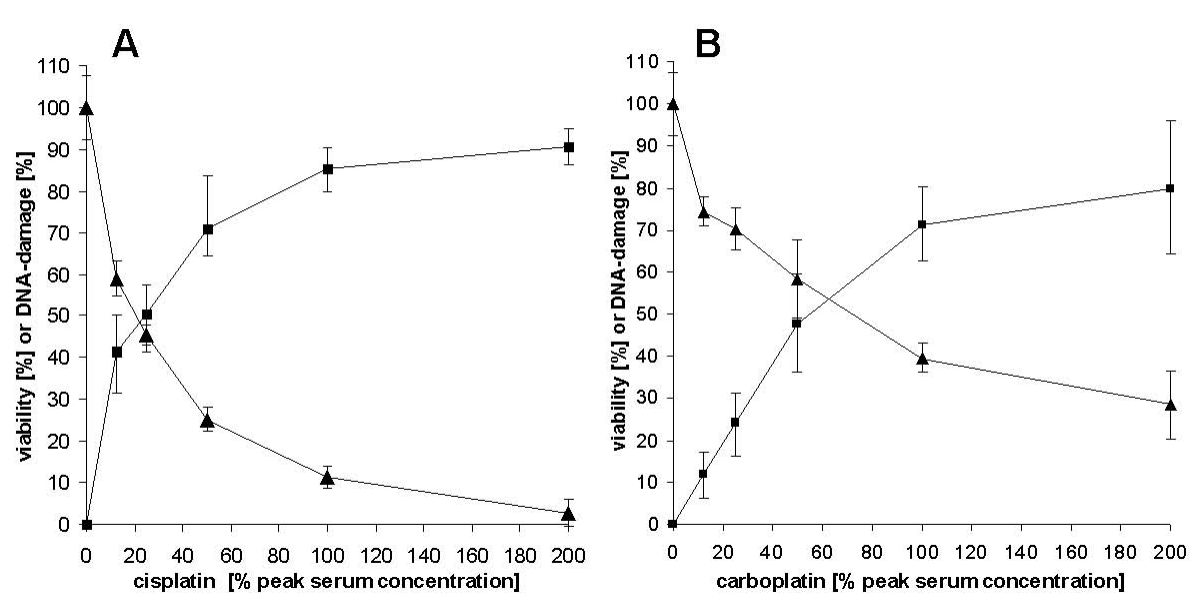
**Concentration dependent DNA damage (black square) presented as median with first and third quantile, and cytotoxicity (black triangle) presented as mean with standard deviation, induced by cisplatin (A), or carboplatin (B)**. DNA damage was measured after a 24 h incubation, cytotoxicity after a 7 day incubation with the cytostatics. MMS alone induced a tail moment ranging from 80-90. The platinum compounds alone induced no tail moments.

The concentration dependence of DNA damage and cytotoxicity induced by the platinum compounds is shown Fig. 2. The DNA damage after a 24 h incubation with cis- (Fig. 2A) and carboplatin (Fig. 2B) was compared with the cytotoxicity obtained after a 7 day treatment. Genotoxicity was detected at the lowest concentration measured (12,5% peak serum concentration) and up. At 100% peak serum concentration the curves show a saturation level with 85% DNA damage for cisplatin and 70% for carboplatin. A doubling of the concentration did not significantly enhance DNA damage

This may be explained by reaching the upper detection limit of 100%. Cell viability continuously decreased to 3% at 200% peak serum concentration for cisplatin and to 30% for carboplatin.

**Figure 3:**
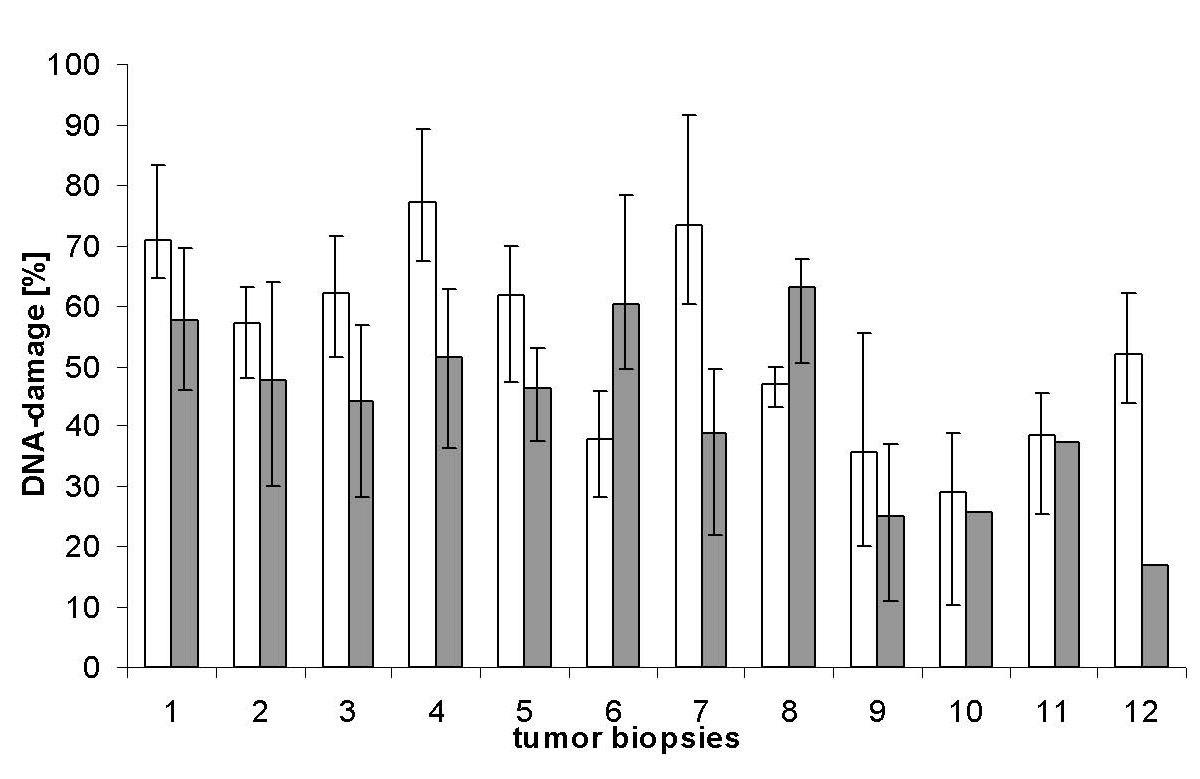
**DNA damage by a 24 hour incubation of cisplatin (white bars) and carboplatin (dark bars) in ovarial cancer cells from 12 biopsies presented as median with first and third quartile**. The platinum concentration was 50% peak serum. MMS alone induced a tail moment ranging from 80-90. The platinum compounds alone induced no tail moments.

After we had optimized the basic parameters measuring the DNA damage induced by cis- and carboplatin 12 biopsies of patients with ovarian cancer were examined. The results of a 24 hour treatment with 50% peak serum concentration of cis- or carboplatin are shown in Fig. 3. The DNA damaging potential of cisplatin was in 11 biopsis higher than that of carboplatin. The cellular sensitivities to cis- or carboplatin differed by a factor of about 3. Similar differences were also observed measuring the reduction of viability (data not shown).

**Figure 4:**
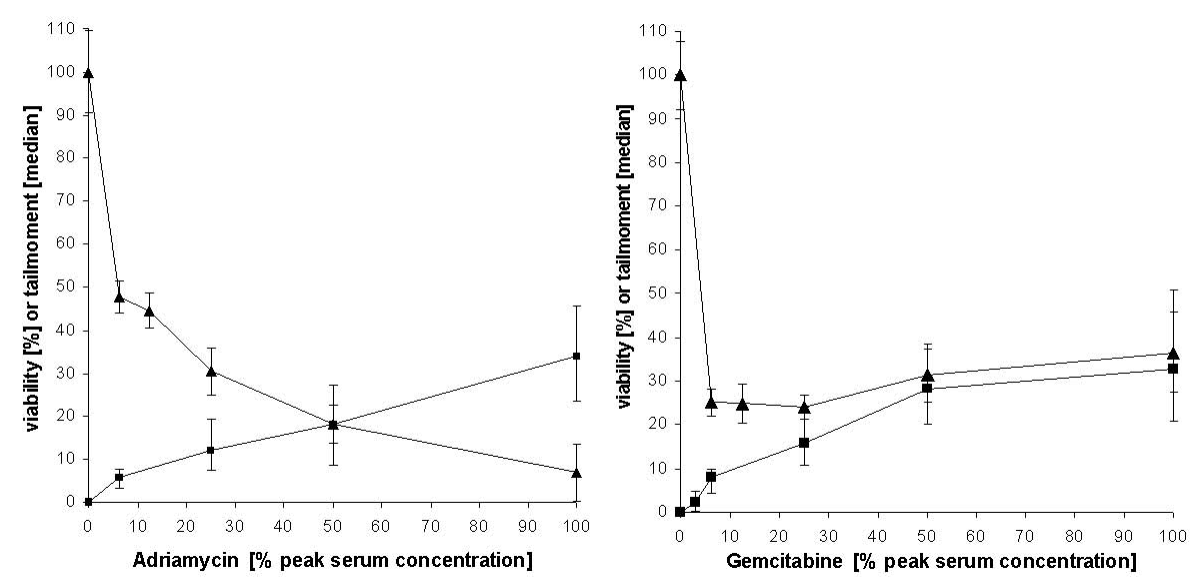
**Concentration dependent DNA damage (black square) presented as median with first and third quartile, and cytotoxicity (black triangle) presented as mean with standard deviation, induced by adriamycin (A) and gemcitabine (B)**. DNA damage was measured after a 24 h incubation, cytotoxicity after a 7 day incubation.

**Figure 5:**
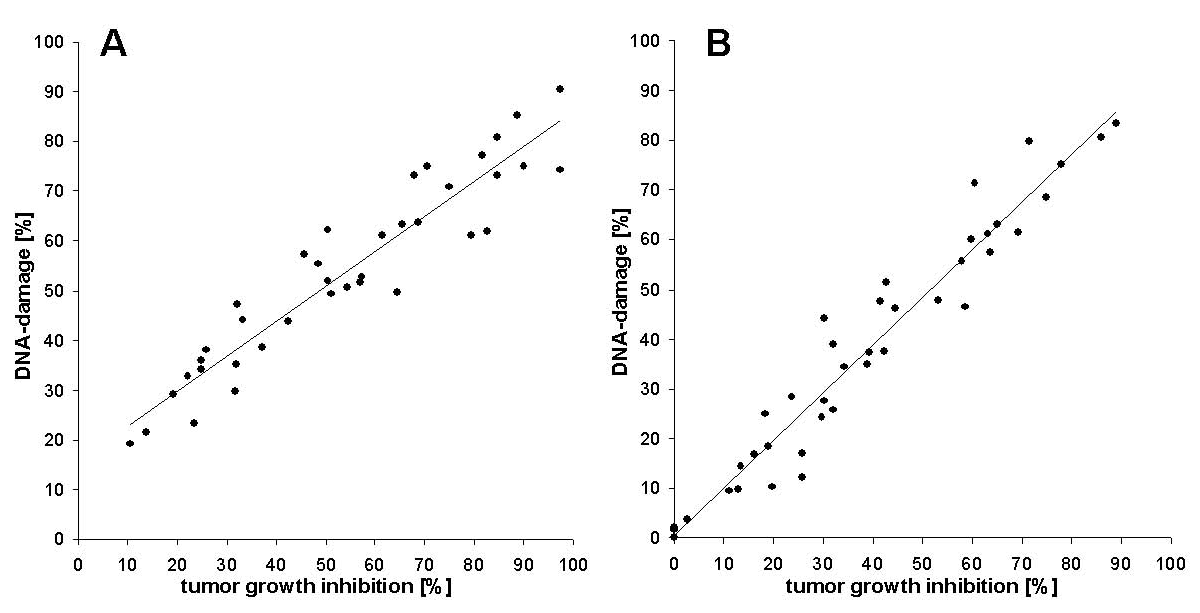
**Correlation of DNA damage in ovarian cancer cells induced by cisplatin (A) (24 h treatment; r = 0,9422), or carboplatin (B) (24 h treatment; r = 0,9711) and cytotoxicity (induced by a 7 day treatment)**. Data were obtained from 12 biopsies.

In addition to the platinum compounds the DNA damaging effects of adriamycin and gemcitabine were measured. Both cytostatics also provoked DNA damage after a 24 h incubation in a concentration dependent manner (Fig. 4 and 5). While in increasing concentrations adriamycin enhanced cytotoxicity (Fig. 4A) gemcitabine did not (Fig. 4B). At the lowest concentration measured (6.25% peak serum concentration) gemcitabine reduced cell viability to 25% after a 7 day incubation. This was not further reduced by higher concentrations of gemcitabine.

**Figure 6:**
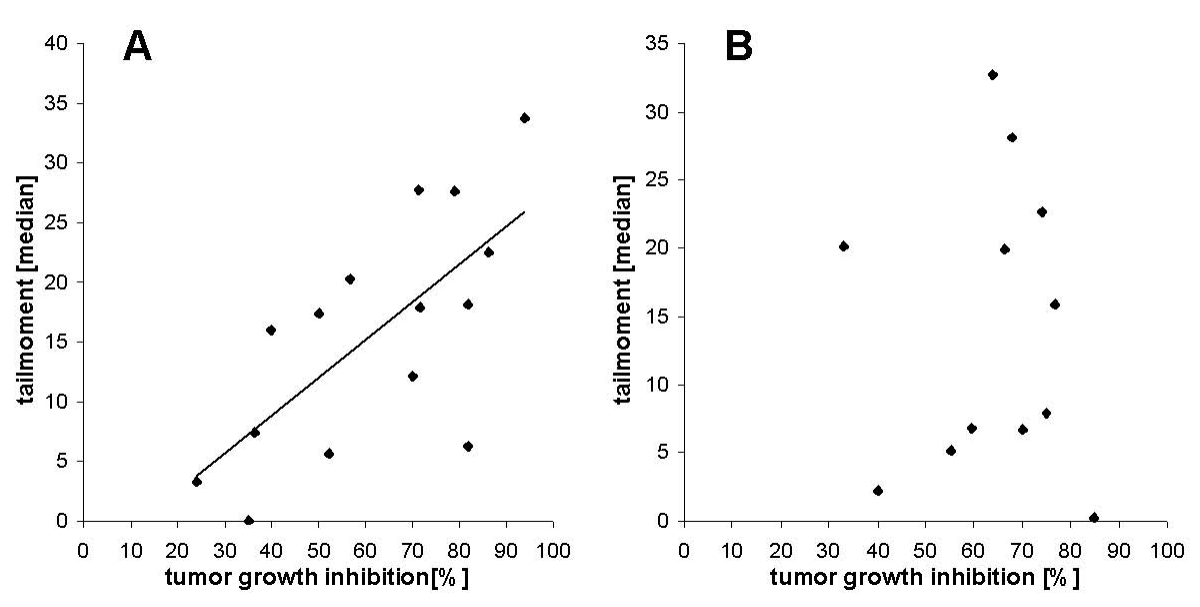
**Correlation of DNA damage induced by adriamycin (A) (24 h treatment; r = 0,6918), or gemcitabine (B) (24 h treatment) and cytotoxicity (induced by a 7 day treatment)**. Data were obtained from 7 biopsies.

If DNA damage induced by cytostatics would be the main cause of their cytotoxicity, a good statistical correlation of DNA damage and viability would be expected. For cis- and carboplatin the correlation of DNA damage (1 day treatment) and cell viability (7 day treatment), measured in concentrations ranging from 6,25%- to 200% peak serum concentration, is shown in Fig. 5. A good correlation r^2 ^= 0.942 for cisplatin (Fig. 5A) and r^2 ^= 0.971 for carboplatin (Fig. 5B) from 12 biopsies was obtained. For adriamycin and gemcitabine only 7 biopsies were examined. Here the correlation was extremely low (Fig. 6A) with r = 0,691 for adriamycin, and was nonexistent for gemcitabine (Fig. 6B).

## Discussion

Carbo- and cisplatin are generally used in combination with paclitaxel in the first line standard chemotherapy of ovarian carcinomas. In the second line therapy these platinum compounds are often administered again without knowing if the cancer cells are resistant, or of lowered sensitivity toward those chemotherapeutics. From 12 biopsies tested in this study a difference in the platinum sensitivities up to a factor of 3 was measured. It is therefore of great importance to know in advance the sensitivity of the individual cancer cells before a second line therapy is started.

To determine the sensitivity of ovarian cancer cells toward platinum compounds several methods were described like measurement of cell viability [8-12,17,18], of predictive marker genes [19], relevant genomics [19,20], or proteomics [21]. From these methods the determination of cell viability is the most often used test which is also commercially available [22]. Of the cell viability assays for studying in vitro chemosensitivity the FDA assay was favoured here over the ATP assay because only in the FDA assay dead and live cells can be visualized, so that possible subclones of resistant cells are recognized and eliminated. For both cytotoxicity assays a correlation with the clinical outcome was shown [8-12].

A critical point of the viability assays is the long *ex vivo *incubation of 5-6 days not knowing if the behaviour of the cells toward chemotherapeutics is changing. We used the comet assay to measure genotoxicity in 2 days. Within this time interval cell transformations are unlikely.

Martelli [23] observed DNA damages already after a 4 hours incubation with platinum compounds in therapeutic relevant concentrations. The 24 h incubation in our experiments yielded more than 75% of DNA damage of the maximal DNA damage (obtained after 7 days incubation). This demonstrates the high sensitivity of the comet assay. Cisplatin was more geno- and cytotoxic than carboplatin. This is in accordance with data from literature [23,24]. Significant cytotoxicity was not observed before day 2 of incubation with a 100% peak serum concentration cisplatin and 4 day for carboplatin. The negligible cytotoxicity after a one day incubation is important to avoid false positive results in the comet assay. It was shown that cytotoxicity > 30% can induce comet formation also by non-genotoxic compounds [25,26].

To correlate DNA damage with cytotoxicity the optimum of incubation time of each assay was used. This was a 24 h incubation for measuring genotoxicity and a 7 day incubation for cytotoxicity. It may be argued that individual DNA repair phenomena were not completely considered during the short test period of one day although the individual DNA repair may play a role in the chemosensitivity of the patients toward the platinum compounds. It was manifold described that the efficacy of platinum compounds in ovarian cancer is influenced by many factors including decreased accumulation or enhanced detoxification [27,28]. These factors are covered by a 24 h genotoxicity measurement. It was shown that the clinical outcome of patients with ovarian carcinoma correlates with Cu-transporting uptake of ATPase [29] and P-glycoprotein expression [30], both responsible for platinum uptake. Furthermore, a correlation of clinical data and deactivation of platinum compounds within the cell via glutathione S transferase pi [31,32] or metallothionein [33] was observed. The sum of these factors may superimpose the influence of DNA repair. Therefore, individual DNA repair (which in our test is only comprised of the repair during the 24 h incubation time) seems to play a minor role in the correlation of DNA damage and cytotoxicity. If the individual DNA repair would be that important, we could not explain the observed correlation of DNA damage and cytotoxicity.

The correlation analysis revealed a strong correlation between DNA damage and cell viability for cis- and carboplatin. This result leads to two conclusions. First, both assays independent of each other were suitable for determining the sensitivity of ovarian cancer cells towards the platinum compounds. Secondly DNA damage must be the main if not the only cause for cytotoxicity as described by other authors [2]. Reed et al. [1] demonstrated that the formation of cis- and carboplatin-DNA adducts in leucocytes of ovarian cancer patients correlated well with the clinical response. This supports our findings that measurement of DNA damage induced by the platinum compounds reflects the response to the chemotherapy.

In contrast to the platinum compounds, DNA damage induced by gemcitabine or adriamycin did either not correlate with the cytotoxicity, or the correlation was low. For both cytostatics multiple molecular effects are known. Radical formation, inhibition of several enzymes like topoisomerase I and II, helicases and some polymerases were described for adriamycin [34-36]. Gemcitabine influences several metabolic ways in the cell like DNA synthesis, RNA synthesis and DNA repair [37]. All these adverse effects will influence cytotoxicity. Therefore, DNA damage by adriamycin and gemcitabine is only one effect of many others. Thus, a correlation of cytotoxicity with only one of these parameters, like the DNA damage, could not be observed. To use the comet assay for cellular chemosensitivity testing of other chemotherapeutics, like oxazophosphorines, nitrosoureas, actinomycin D, or mitomycin C, it is at first necessary to verify the positive correlation between cyto- and genotoxicity for each compound, individually, as shown here for cis- and carboplatin.

## In conclusion

The comet assay is a suitable method for determining the sensitivity of ovarian cancer cells toward cis- and carboplatin. The comet assay has some advantages over the often used in vitro chemosensitivity tests measuring cytotoxicity. In the comet assay described here, the results are obtained two days after receiving the cancer biopsy, in contrast to 5-6 days in the ATP cytotoxicity test [16]. Therefore, changes in the behaviour of the cancer cells ex vivo can be minimized. Another advantage is the low cell number needed for the comet assay. It is possible to measure only one concentration (50% peak serum concentration), while in the cell viability assay one has to determine a dose effect curve with four parallel samples per concentration [21]. Using 96 well plates with 3.000 cells per well, about 18.000 cells were needed for the comet assay. It means that needle biopsies are sufficient to determine the sensitivity of these cancer cells. This is important especially for small recurrent cancer biopsies and metastases where only needle biopsies are available.

## Competing interests

The authors declare that they have no competing interests.

## Authors' contributions

FTU participated in the design of the study, carried out the molecular studies and performed the statistical analysis. HAK has been involved in the coordination of the study and helped to draft the manuscript. GT isolated cancer cells and characterized cancer biopsies. RLdW participated in the coordination of the study and interpretation of the data. IW conceived of the study, participated in its design and drafted the manuscript. All authors read and approved the final manuscript.

## Pre-publication history

The pre-publication history for this paper can be accessed here:

http://www.biomedcentral.com/1471-2407/9/359/prepub
